# Cognitive and Psychological Sequelae of COVID-19: Age Differences in Facing the Pandemic

**DOI:** 10.3389/fpsyt.2021.711461

**Published:** 2021-09-16

**Authors:** Maria Devita, Elisa Di Rosa, Pamela Iannizzi, Sara Bianconi, Sara A. Contin, Simona Tiriolo, Nicol Bernardinello, Elisabetta Cocconcelli, Elisabetta Balestro, Annamaria Cattelan, Davide Leoni, Daniela Mapelli, Biancarosa Volpe

**Affiliations:** ^1^Department of General Psychology (DPG), University of Padua, Padua, Italy; ^2^U.O.C. Clinical Psychology, Hospital of Padua, Padua, Italy; ^3^Department of Cardiac, Thoracic, Vascular Sciences and Public Health, University of Padova, Padua, Italy; ^4^Infectious Disease Unit, Hospital of Padua, Padua, Italy

**Keywords:** COVID-19, pandemic, cognitive effects, post-traumatic stress disorder, age differences

## Abstract

Literature about the novel Coronavirus (COVID-19) is currently focusing on the potential cognitive and neuropsychiatric sequelae observed in individuals receiving intensive care unit (ICU) treatments. The aim of the present study is to evaluate the differences in cognitive and psychological sequelae of COVID-19 between younger and older adults, regardless of being admitted to the ICU or not. The study involved 299 recovered individuals (from 18 to 90 years old), who underwent a comprehensive cognitive and psychological assessment. Linear regression models were conducted separately for Montreal Cognitive Assessment (MoCA) test and Post-traumatic Stress Disorder Checklist (PCL) scores to investigate the effect of socio-demographic and clinical characteristics on them. Separate linear regression models were then applied sorting participants by age: younger adults (<65 years) and older adults (≥65 years). In the whole sample, PCL scores were predicted by the intensity of care received, by being intubated, and by the persistence of cough after 1 month after hospitalization. Only age had instead an effect on cognition. In younger adults, PCL scores were predicted by the presence of neurological symptoms, by the intensity of care received, and by being intubated; MoCA scores were only predicted by the intensity of care received. No significant associations were found in older adults. Psychological negative effects of the COVID-19 pandemic particularly affect individuals under 65 years old, who also subjectively report cognitive sequelae associated with the infection. Individuals over 65 years old, instead, seem to be free from psychological and cognitive difficulties due to COVID-19.

## Introduction

The entire world is currently in the middle of an unprecedented and delicate historical phase: the spread of the novel Coronavirus (SARS-CoV-2), a very contagious virus that causes a severe acute respiratory syndrome (COVID-19). Symptoms of COVID-19 are variable, but often include fever, cough, fatigue, breathing difficulties, and loss of smell and taste. As recently reviewed by Asadi-Pooya and Simani (1), COVID-19 has also been associated with neurological manifestations (e.g., febrile seizures, convulsions, change in mental status, and encephalitis); upon nasal infection, coronavirus enters the central nervous system (CNS) through the olfactory bulb, causing inflammation, and demyelination (2). A CNS involvement made clinicians and researchers question potential cognitive and psychological sequelae associated with COVID-19 infection. Current literature regarding the specific cognitive impact of virus-related CNS/neurological disease is still quite limited. A recent study showed that 15 of 45 patients diagnosed with COVID-19 exhibited a dysexecutive syndrome consisting of inattention, disorientation, and difficulties organizing response to command during hospitalization at the intensive care unit (ICU) (3). Accordingly, other studies also highlighted the presence of cognitive difficulties mainly in working memory, divided attention, and processing speed (4). Beside cognitive sequelae, there is also evidence of persistent neuropsychiatric disorders associated with COVID-19 (5). Not only were delirium, anxiety, depression, and insomnia found to characterize the acute phases of infection, but they were also reported in follow-up studies ranging from 6 weeks to 39 months post contagion (6). So far, special attention has been understandably paid to individuals with COVID-19 who were hospitalized in ICUs. In fact, the permanence in these units is commonly associated with the onset of cognitive deficits (7, 8), post-traumatic stress disorder (PTSD), depression, and generalized anxiety (9–11). However, given the evidence that COVID-19 impacts CNS functioning, it is becoming crucial to understand the impact of this virus on cognitive and psychological daily functioning, also among those individuals who, regardless of being admitted to the ICU or not, still have contracted the infection. Therefore, the aim of the present study is to evaluate the differences in cognitive and psychological sequelae of COVID-19 between younger and older recovered adults assessed within 1 month after the last negative nasopharyngeal swab test. The hypotheses were that, on the one hand, younger adults could suffer the most from a psychological point of view, because of the social and affective restrictions imposed by the pandemic, and the consequent limitation of their personal freedom. On the other hand, older adults were hypothesized to suffer the most from a cognitive point of view, because of the well-known deleterious effects of social and environmental deprivation on neuropsychological efficiency.

## Materials and Methods

### Participants

This is a retrospective, observational clinical study carried out at the Azienda Ospedale-Università of Padova (Italy) that involved the Hospital Psychology, the Infectious Diseases, and the Pneumology Operational Units. Data on 299 recovered individuals previously diagnosed with COVID-19 were collected during post-hospitalization pneumological and infectious follow-ups, occurring 1 month after the last negative nasopharyngeal swab test. Inclusion criteria were previous positivity to COVID-19 virus, a previous consultation, and/or hospitalization at the Infectious Diseases Unit (Azienda Ospedale-Università of Padova) and age between 18 and 90 years. Exclusion criteria were being non-Italian native speakers and the presence of important pre-existing physical/sensory/psychiatric illness that did not permit the administration of psychometric tests. Individuals who were not able to give their written informed consent to participate to the study were also excluded. Information about socio-demographic characteristics, proximate pathological anamnesis due to COVID-19 (kind and duration of symptoms, hospitalization and treatments received), remote pathological anamnesis and comorbidities, and general lifestyle habits (physical activity, smoking, alcohol consumption, and nutrition) were obtained retrospectively from medical records. All participants provided written informed consent before entering the study. The protocol was in accordance with the Helsinki Declaration on human rights and was approved by the Ethics Committee of the Padova University Hospital (Prot. Number 0014424).

### Assessment

Every participant was asked to attend a psychological assessment session. The present and premorbid patient's cognitive and psychological well-being was explored by means of a preliminary semi-structured clinical interview, after which a list of standard cognitive and psychological assessment tests was administered (see Supplementary materials). In the present study, the following tests, in their Italian validation, will be considered:

- Montreal Cognitive Assessment [MoCA. (12)], which evaluates global cognitive functioning through 30 items assessing: short-term memory; visuospatial abilities by clock drawing and a cube copy task; executive functioning through an adaptation of Trail Making Test Part B, phonemic fluency, and verbal abstraction; attention, concentration, and working memory by means of target detection, serial subtraction, digits, and digits backward; language *via* confrontation naming with low-familiarity animals and repetition of complex sentences; and orientation to time and place (12). A total score is obtained by summing all the items, and the authors recommend a clinical cutoff score of 26 [although this value may vary accordingly to age and education of the population for which it is validated; for the Italian population, see, for example, (13)].
- Post-traumatic Stress Disorder Checklist [PCL; (14)], which assesses the presence and severity of PTSD symptoms according to the Diagnostic and Statistical Manual of Mental Disorders-5 (DSM-5) PTSD criteria. PCL is a 20-item self-report measure applied for a variety of purposes, including monitoring symptom change during and after treatment, screening individuals for PTSD, and making a provisional PTSD diagnosis. Responders are asked to rate how bothered they have been by each item (e.g., “Repeated, disturbing, and unwanted memories of the stressful experience?”) in the past month on a five-point Likert scale ranging from 0 (“not at all”) to 4 (“extremely”). Items are summed to provide a total score. A PCL cut-point of 33/80 appears to be (14) a reasonable value to use for provisional PTSD diagnosis.

## Statistical Analyses

Linear regression models were applied separately for cognitive and psychological profiles, in order to investigate the effect of a series of predictors (i.e., age, presence and kind of symptoms related to COVID-19, intensity of care) on, respectively, MoCA scores and PCL (dependent variables). As well as for the whole sample, separate linear regression models were then applied sorting participants by age: adults (<65 years) and older adults (≥ 65 years), in order to investigate the effect of predictors on dependent variables in these two different population groups. Statistical analyses were carried out using SPSS software, version 21 (IBM Corp. 2012).

## Results

Descriptive characteristics of the sample as a whole and sorted by age are shown in Table 1.

**Table 1:** Shows descriptive characteristics of the sample as a whole and sorted by age.

	**Total sample**	**Age <65**	**Age ≥ 65**	***p***
	**(*N* = 299; *F* = 135)**	**(*N* = 209; *F* = 98)**	**(*N* = 90; *F* = 37)**	
Age Mean (±SD)	57.11 (14.58)	49.89 (10.74)	73.88 (5.92)	<0.001
Education Mean (±SD)	12.51 (4.70)	13.47 (4.07)	10.31 (5.31)	<0.001
**Subjective reports**				
Cognitive impairment BC	82 (27.30%)	42 (20.10%)	40 (44.40%)	<0.001
Cognitive impairment AC	92 (30.70%)	68 (32.50%)	24 (26.70%)	0.57
MoCA total score Mean (±SD)	24.88 (3.53)	26.11 (2.34)	22.29 (4.16)	<0.001
TMT-B		0.911 (0.286)	0.696 (0.463)	<0.001
Copy cube		0.893 (0.310)	0.600 (0.493)	<0.001
Draw clock		2.792 (0.449)	2.531 (0.726)	<0.001
Naming		2.958 (0.297)	2.741 (0.519)	<0.001
Digit span		1.685 (0.549)	1.407 (0.628)	<0.001
Tapping		0.952 (0.214)	0.877 (0.331)	0.030
Subtractions		2.881 (0.376)	2.654 (0.674)	<0.001
Repetition		1.560 (0.533)	1.321 (0.649)	0.002
Fluency		0.667 (0.473)	0.494 (0.503)	0.009
Abstraction		1.774 (0.498)	1.420 (0.630)	<0.001
Delayed recall		3.089 (1.379)	1.802 (1.461)	<0.001
Category cue		0.647 (0.829)	1.111 (0.987)	<0.001
Multiple choice cue		0.892 (1.012)	1.222 (1.151)	0.022
Orientation		5.958 (0.200)	5.901 (0.300)	0.077
PCL Mean (±SD)	31.32 (12.12)	31.76 (12.68)	30.12 (10.60)	0.28
Neurological symptoms *N* (%)	172 (57.30%)	126 (60.30%)	46 (51.10%)	0.11
**Comorbidities**				
Cardiovascular *N* (%)	121 (40.30%)	59 (28.20%)	62 (68.90%)	<0.001
Pulmonary *N* (%)	48 (16%)	31 (14.80%)	17 (18.90%)	0.18
Autoimmune *N* (%)	42 (14%)	29 (13.90%)	13 (14.40%)	0.84
Dyslipidemia *N* (%)	93 (31%)	51 (24.40%)	42 (46.70%)	<0.001
Oncological *N* (%)	36 (12%)	13 (6.20%)	23 (25.60%)	<0.001
Days of Hospitalization Mean (±SD)	11.8 (10.3)	10.1 (9)	15.4 (12.1)	<0.001
Intensity of care during hospitalization				0.08
AA *N* (%)	113 (37.70%)	91 (43.50%)	22 (24.40%)	
Oxygen mask *N* (%)	117 (39%)	75 (35.90%)	42 (46.70%)	
HFNC *N* (%)	11 (3.70%)	6 (2.90%)	5 (5.60%)	
NIV *N* (%)	19 (6.30%)	10 (4.80%)	9 (10%)	
IOT *N* (%)	20 (6.70%)	14 (6.70%)	6 (6.7%)	
Intubated *N* (%)	22 (7.30%)	15 (7.20%)	7 (7.80%)	
**Sequelae associated with COVID-19**				
Smell/taste loss	19 (7.51)	14 (8.04)	5(6.3)	0.63
Muscular attractions	39 (15.41)	31 (17.81)	8 (10.12)	0.11
Hair loss	22 (8.69)	16 (9.19)	6 (7.59)	0.67
Difficulty concentrating	15 (5.92)	11 (6.32)	4 (5.06)	0.69
Insomnia	7 (2.76)	6 (3.44)	1 (1.26)	0.32
Migraine	10 (3.95)	9 (5.17)	1 (1.26)	0.14
Gastrointestinal disorders	6 (2.37)	5 (2.87)	1 (1.26)	0.43
Cough	19 (7.51)	14 (8.04)	5 (6.32)	0.63

*AA, no oxygen supply; HFNC, high flow nasal cannula; NIV, non-invasive ventilation; IOT, intensive oxygen therapy; MoCA, Montreal Cognitive Assessment; PCL, Post-traumatic stress disorder checklist; BC, before COVID-19 infection; AC, after COVID-19 infection*.

Continuous variables are expressed as mean and standard deviation (M ± SD); categorical variables are expressed as numerosity and frequencies (*n*, %).

As reported, the mean age of participants was 57.1 ± 14.5 and the majority were males (54.7%). Most of the sample is composed of individuals who used to smoke (28.7%) and who suffered from cardiovascular diseases (40.3%). Of the whole sample, 7.3% needed to be intubated during hospitalization; among these individuals, the majority was <65 years. Higher scores at the MoCA test are observed in younger adults compared to older adults (higher cognitive functioning). At the same time, a higher level of PTSD (higher PCL scores) was detected in younger adults, when compared with the older ones. Of the whole sample, 30.7% suffered from cognitive frailties (i.e., difficulties in memory and/or in focusing attention) after COVID-19 infection.

### Linear Regression Models for the Whole Sample

Two separate linear regression models were applied in order to investigate potential predictors of cognitive (MoCA scores) and psychological (PCL scores) frailties due to COVID-19 infection. See Table 2.

**Table 2:** Shows linear regression models for the whole sample.

	**MoCA**			**PCL**		
	***B* (SE)**	***t***	***p***	***B* (SE)**	***t***	***p***
Age	−0.45 (0.01)	−7.03	**<0.001**			
Intensity of care	−0.06 (0.25)	−0.68	0.49	−0.20 (1.05)	−2.09	**<0.05**
Intubation	−0.01 (1.08)	−0.20	0.83	−0.18 (4.40)	−2.08	**<0.05**
Persistent cough	−0.03 (0.89)	−0.50	0.61	0.25 (3.53)	3.67	**<0.001**
Smell/taste loss	−0.01 (0.53)	−0.23	0.81	0.03 (1.99)	0.39	0.69
Muscular attractions	0.08 (0.54)	1.25	0.21	0.02 (2.08)	0.33	0.73
Hair Loss	−0.02 (0.68)	−0.31	0.75	−0.06 (8.32)	−0.89	0.37
Difficulty concentrating	−0.06 (1.03)	−0.79	0.42	0.06 (9.44)	0.74	0.45
Insomnia	0.03 (1.45)	0.40	0.68	0.13 (4.93)	1.79	0.07
Migraine	−0.02 (1.22)	−0.36	0.71	0.10 (4.54)	1.48	0.13
Gastrointestinal disorders	0.03 (1.60)	0.58	0.56	−0.06 (7.63)	−0.77	0.44
Duration of hospitalization	−0.27 (0.03)	−2.89	**<0.05**	−0.02 (0.10)	−0.22	0.81

*MoCA, Montreal Cognitive Assessment; PCL, Post-traumatic stress disorder checklist; SE, standard error*.

*The bold values means p < 0.05*.

#### Cognitive Profile

Results indicated that the only significant MoCA score predictors are age, with cognitive performance decreasing as age increases, and the duration of hospitalization. The presence of neurologic symptoms [β = 0.08 (0.41), *p* = 0.19], the intensity of care received [β = −0.06 (0.25), *p* = 0.49], being intubated during hospitalization [β = −0.01 (1.08), *p* = 0.83], the presence of PTSD [β = 0.03 (0.01), *p* = 0.57], and other alterations due to COVID-19 infection (i.e., muscular alterations, hair loss, concentration difficulty, insomnia, headache, gastrointestinal disorders, and cough) do not have any significant role in predicting cognitive performance.

#### Psychological Profile

Results also showed that PCL scores are instead predicted by the intensity of care received by being intubated during hospitalization and by the persistence of cough 1 month after hospitalization (see Table 2), while the presence of neurologic symptoms [β = 0.11 (1.68), *p* = 0.09] or of other alterations due to COVID-19 infection (i.e., muscular alterations, hair loss, concentration difficulty, insomnia, headache, gastrointestinal disorders, and cough) did not have any significant predictive role.

### Linear Regression Models for the Sample Sorted by Age

Two separate linear regression models were applied in order to investigate potential predictors of cognitive (MoCA scores) and psychological (PCL scores) frailties due to COVID-19 infection for “adults” (<65 years) and for “older adults” (≥ 65 years) separately. See Table 3.

**Table 3:** Shows linear regression models for the sample sorted by age.

	**MoCA**						**PCL**					
	**Age <65**			**Age ≥65**			**Age <65**			**Age ≥65**		
	***B* (SE)**	***t***	***p***	***B* (SE)**	***t***	***p***	***B* (SE)**	***t***	***p***	***B* (SE)**	***t***	***p***
Intensity of care	−0.30 (0.22)	−2.87	**<0.01**	0.03 (0.55)	0.18	0.85	−0.24 (1.26)	−2 to −22	**<0.05**	0.02 (1.74)	0.10	0.91
Neurological symptoms	0.11 (0.40)	1.34	0.18	0.15 (1.14)	1.09	0.28	0.18 (2.10)	2.29	**<0.05**	0.01 (3.05)	0.12	0.90
Intubation	−0.09 (1.00)	−0.81	0.41	0.13 (3.27)	0.57	0.56	−0.20 (5.43)	−1.95	**<0.05**	−0.03 (8.53)	−0.17	0.86
Persistent cough	−0.22 (0.96)	−2.4	**<0.01**	0.18 (2.49)	1.17	0.24	0.17 (4.44)	2.10	**<0.05**	0.20 (6.63)	1.30	0.19
Smell/taste loss	0.08 (0.69)	0.95	0.34	−0.17 (1.94)	−1.27	0.20	0.09 (3.76)	1.20	0.23	0.03 (5.18)	0.28	0.78
Muscular attractions	−0.08 (0.50)	−0.90	0.37	0.23 (1.92)	1.39	0.16	0.05 (2.51)	0.72	0.46	−0.13 (5.76)	−0.74	0.46
Hair loss	−0.02 (0.66)	−0.24	0.80	−0.03 (2.54)	−0.24	0.80	−0.03 (3.35)	−0.38	0.70	0.21 (6.76)	1.31	0.19
Difficulty concentrating	−0.07 (0.93)	−0.66	0.50	−0.06 (4.46)	−0.24	0.80	0.25 (4.60)	2.55	**<0.01**	−0.12 (13.08)	−0.41	0.67
Insomnia	0.03 (1.25)	0.34	0.73	0.13 (6.86)	−0.58	0.56	−0.02 (6.31)	−0.26	0.79	0.37 (19.11)	1.59	0.11
Migraine	−0.09 (1.03)	−1.08	0.28	0.03 (6.30)	0.15	0.87	0.11 (5.2)	1.35	0.17	−0.10 (18.01)	−0.49	0.62
Gastrointestinal disorders	0.05 (1.42)	0.55	0.58	−0.12 (7.25)	−0.52	0.60	−0.09 (7.11)	−1.12	0.26	0.08 (20.60)	0.34	0.73
PCL	0.01 (0.01)	0.95	0.82	0.07 (0.05)	0.53	0.59						
Duration of hospitalization	0.08 (0.03)	0.06	0.94	−0.26 (0.05)	−1.55	0.12	−0.13 (0.18)	−1.26	0.20	0.16 (0.14)	0.89	0.37

*MoCA, Montreal Cognitive Assessment; PCL, Post-traumatic stress disorder checklist; SE, standard error*.

*The bold values means p < 0.05*.

#### Cognitive Profile

in younger adults, MoCA scores are predicted only by the intensity of care received during hospitalization, while the presence of neurologic symptoms [β = 0.13 (0.38), *p* = 0.08], being intubated during hospitalization [β = −0.11 (0.98), *p* = 0.26], or the presence of PTSD [β = 0.01 (0.01), *p* = 0.82] does not predict cognitive performance in this population. In older adults, instead, MoCA scores are not predicted by any of the potential predictor analyzed.

#### Psychological Profile

in younger adults, PCL scores are predicted by the presence of neurological symptoms related to COVID-19, by the intensity of care received, by being intubated during hospitalization, by persistent cough, and by experiencing difficulties in concentrating. In older adults, none of the variables considered predict PCL scores.

## Discussion

The present study aimed at evaluating the differences in cognitive and psychological sequelae of COVID-19 between younger and older recovered adults, assessed within 1 month after the last negative nasopharyngeal swab test. As a matter of fact, the evidence so far has highlighted how people suffered from cognitive and psychological sequalae after infection mainly as a consequence of ICU stay. Instead, a characterization of cognitive and psychological profiles of the overall population infected by this highly contagious virus is still largely undefined. Our study suggests that the cognitive profile of individuals who had COVID-19 is unscathed by the presence of neurologic or other symptoms, by the intensity of care received, and by being intubated that, instead, predicts PTSD. Our results confirm that cognition is negatively influenced by the duration of hospitalization, corroborating previous findings that showed detrimental effects of hospitalization on cognition (15). A similar pattern is observable also by sorting the sample by age: cognitive performance is predicted in younger adults only by the intensity of care received, while no other variables were associated with MoCA scores. Cognitive profile of older adults, conversely, seem to be not even influenced by the intensity of care, and none of the variables examined predicted MoCA scores. The psychological profile (i.e., the presence of PTSD) of the younger adults is, instead, confirmed to be predicted by the presence of neurological symptoms related to COVID-19, by the intensity of care received, and by being intubated during hospitalization. Also, in this case, older adults seem to be unharmed by PTSD associated with COVID. Therefore, our results suggest that there is no clear evidence for cognitive impairment related to COVID-19; rather, it seems that PTSD is the main outcome following the infection. In our study, individuals under 65 years old are those who mostly suffer from the deleterious psychological effects of the pandemic and that more older adults are influenced not only by the actual intensity of cure received, but also by the sole presence of neurological symptoms. Accordingly, it is interesting to note how self-reported cognitive impairments after infection are increased among adults compared to older adults (respectively, 20% before COVID, vs. 32.5% after COVID, against 44.4% before COVID, vs. 26.7% after COVID).

A possible explanation of this result is that younger individuals are less used to experiencing some sort of diseases; on the contrary, older individuals may have already faced pathologies and are more “familiar” with an illness context. Another possible explanation, also corroborated by previous findings (16), is that older adults are supposed to be more resilient, especially with respect to emotional regulation ability and problem solving, than younger individuals in facing the pandemic. COVID-19 has certainly undermined everyone's resilience (17, 18); however, as highlighted also by Losada-Baltar et al. (19), lower chronological age is one of the main negative predictors of psychological well-being during the COVID-19 outbreak. It is possible that not only the infection itself, but also the social restrictions, and all the daily routine upheavals [including, in some cases, occupational and salary changes, see (20)] have particularly affected adults, rather than older adults, who are now paying the highest psychological and (subjective) cognitive price of the pandemic.

### Limitations and Strengths

Some limitations should be certainly acknowledged. First of all, an assessment of psychological and cognitive profiles of the general population before pandemic is lacking, and a rigorous analysis of changes due to COVID-19 on these variables was not achievable. Although we tried to compensate for this weakness by asking participants about their “previous” psychological and cognitive well-being, we still acknowledge this aspect as the main limitation of our study. Furthermore, the sample size sorted by age is not completely well-matched: adults are more numerous than older adults. Although it is known that COVID-19 particularly affects the latter, it is also sadly true that most of them died during hospitalization or at home. Therefore, a potential age bias should be considered in this as well as in other investigations on the topic. In addition, the analyses we performed were carried out on very large age groups; this may have led to neglect specific results related to more restricted and specific population subgroups. Furthermore, we did not collect data about the duration of ICU stay. This represents a limitation of our study since, as found for duration of hospitalization, the effects, on psychological and cognitive well-being, of permanence in ICUs may change according to its extent. Finally, this study lacks a control group composed of individuals who have never contracted the virus. Conversely, the research topic is timely and brings novelty to the COVID-19 literature. In particular, this study contributes to defining psychological and cognitive profiles of the general population affected by the virus and not only of those who received ICU treatment.

## Conclusions

The present study suggests that psychological negative effects of the COVID-19 pandemic particularly affect individuals under 65 years old, who also subjectively report cognitive sequelae associated with the infection. These data, however, are not confirmed by objective neuropsychological assessment. Individuals over 65 years old, instead, seem to be free from psychological and cognitive difficulties due to COVID-19.

## Data Availability Statement

The raw data supporting the conclusions of this article will be made available by the authors, without undue reservation.

## Ethics Statement

The studies involving human participants were reviewed and approved by Regione del Veneto—Azienda Ospedale Università Padova. Comitato Etico per la sperimentazione clinica della provincia di Padova. Prot. N. 0014424. The patients/participants provided their written informed consent to participate in this study.

## Author Contributions

PI, EB, AC, DM, and BV: conceptualization. MD, ED, and DM: methodology. MD and ED: formal analysis and investigation. SB, SC, ST, NB, EC, and DL: data collection. MD: writing—original draft preparation. MD, ED, and PI: writing—review and editing. DM and BV: resources. DM and AC: supervision. All authors contributed to the article and approved the submitted version.

## Conflict of Interest

The authors declare that the research was conducted in the absence of any commercial or financial relationships that could be construed as a potential conflict of interest.

## Publisher's Note

All claims expressed in this article are solely those of the authors and do not necessarily represent those of their affiliated organizations, or those of the publisher, the editors and the reviewers. Any product that may be evaluated in this article, or claim that may be made by its manufacturer, is not guaranteed or endorsed by the publisher.
